# Effects of BIS-MEP on Reversing Amyloid Plaque Deposition and Spatial Learning and Memory Impairments in a Mouse Model of β-Amyloid Peptide- and Ibotenic Acid-Induced Alzheimer’s Disease

**DOI:** 10.3389/fnagi.2019.00003

**Published:** 2019-01-22

**Authors:** Yu Wang, Jia Xia, Mengjun Shen, Yifan Zhou, Zhe Wu, Yuhuan Shi, Jianrong Xu, Lina Hou, Rui Zhang, Zhuibai Qiu, Qiong Xie, Hongzhuan Chen, Yongfang Zhang, Hao Wang

**Affiliations:** ^1^Department of Pharmacology and Chemical Biology, School of Medicine, Shanghai Jiao Tong University, Shanghai, China; ^2^Department of Medicinal Chemistry, School of Pharmacy, Fudan University, Shanghai, China

**Keywords:** Alzheimer’s disease, acetylcholinesterase inhibitor, BIS-MEP, β-amyloid peptide, learning, memory

## Abstract

Alzheimer’s disease (AD) is the main type of dementia and is characterized by progressive memory loss and a notable decrease in cholinergic neuron activity. As classic drugs currently used in the clinic, acetylcholinesterase inhibitors (AChEIs) restore acetylcholine levels and relieve the symptoms of AD, but are insufficient at delaying the onset of AD. Based on the multi-target-directed ligand (MTDL) strategy, bis-(-)-nor-meptazinol (BIS-MEP) was developed as a multi-target AChEI that mainly targets AChE catalysis and the β-amyloid (Aβ) aggregation process. In this study, we bilaterally injected Aβ oligomers and ibotenic acid (IBO) into the hippocampus of ICR mice and then subcutaneously injected mice with BIS-MEP to investigate its therapeutic effects and underlying mechanisms. According to the results from the Morris water maze test, BIS-MEP significantly improved the spatial learning and memory impairments in AD model mice. Compared with the vehicle control, the BIS-MEP treatment obviously inhibited the AChE activity in the mouse brain, consistent with the findings from the behavioral tests. The BIS-MEP treatment also significantly reduced the Aβ plaque area in both the hippocampus and cortex, suggesting that BIS-MEP represents a direct intervention for AD pathology. Additionally, the immunohistochemistry and ELISA results revealed that microglia (ionized calcium-binding adapter molecule 1, IBA1) and astrocyte (Glial fibrillary acidic protein, GFAP) activation and the secretion of relevant inflammatory factors (TNFα and IL-6) induced by Aβ were decreased by the BIS-MEP treatment. Furthermore, BIS-MEP showed more advantages than donepezil (an approved AChEI) as an Aβ intervention. Based on our findings, BIS-MEP improved spatial learning and memory deficits in AD mice by regulating acetylcholinesterase activity, Aβ deposition and the inflammatory response in the brain.

## Introduction

Alzheimer’s disease (AD) is a progressive neurodegenerative disease that slowly destroys memory and cognitive performance in the elderly (De Strooper and Karran, 2016). Although the pathogenesis of AD remains unclear, cholinergic neuron deficits are responsible for the cognitive symptoms of AD. Therefore, acetylcholinesterase inhibitors (AChEIs) can help manage cholinergic neuron recovery and memory loss (Bond et al., 2012; Alzheimer’s Association, 2017). Marketed AChEIs are the mainstream drugs used in the clinic for AD therapy. This intervention may temporarily alleviate symptoms, but rarely exerts long-term effects or slows the pathological process of AD (Pang et al., 1996; Galimberti and Scarpini, 2016).

**Figure 1:**
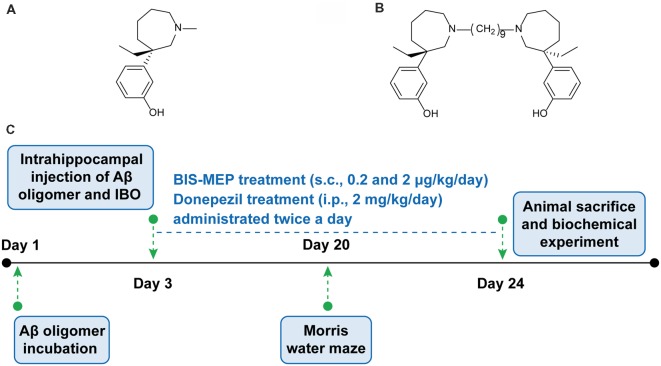
Bis-(-)-nor-meptazinol (BIS-MEP) structure and experimental scheme. Chemical structural of **(A)** (-)-MEP and **(B)** BIS-MEP. **(C)** Schematic of the time course of the BIS-MEP experiment.

Following comprehensive studies of AD, a therapy with synergistic effects on multiple pathogenic factors is a relevant treatment for the disease. Structural studies have revealed that AChE contains a gorge with a catalytic anionic site (CAS) and a peripheral anionic site (PAS). The PAS region accelerates β-amyloid (Aβ) assembly into fibrils (Inestrosa et al., 1996). Based on the dual-binding AChEI strategy, our group has designed and synthesized bis-(-)-nor-meptazinol (BIS-MEP) by linking two (-)-nor-meptazinols (Figure 1A) with a nonamethylene spacer (Figure 1B). As shown in our previous study, one nor-meptazinol moiety of BIS-MEP participates in a hydrogen bond with histidine 447 in the CAS and hydrophobic interactions with tryptophan 86 to disrupt catalytic triad. The other nor-meptazinol moiety binds to tryptophan 286 in the PAS through cation-π and hydrophobic interactions (Paz et al., 2009). The binding mode ensures that BIS-MEP possesses strong AChE inhibitory activity [half maximal inhibitory concentration (IC_50_) = 3.9 ± 1.3 nM, 10,000-fold stronger than (-)-nor-meptazinol] and the capacity to prevent AChE-induced Aβ aggregation (IC_50_ = 16.6 μM; Xie et al., 2008). *In vivo*, the subcutaneous administration of BIS-MEP significantly reverses scopolamine-induced memory impairments in mice (Liu et al., 2013). Since BIS-MEP exhibits multi-target therapeutic potential against AD, an assessment of its effects on more integrated AD models is urgently needed.

Intracerebral injections of Aβ and ibotenic acid (IBO) in mice represent an acute and practical method to mimic AD pathology. A direct infusion of neurotoxic oligomeric Aβ_1–42_ into the hippocampus of wild-type mice replicated the cerebral accumulation of Aβ aggregates, cholinergic dysfunction and cognitive and memory impairments (Van Dam and De Deyn, 2011). Accompanying the Aβ accumulation, activated microglia and astrocytes, the primary inflammatory cells in the central nervous system (CNS), clustered around Aβ plaques and secreted proinflammatory mediators, indicating key roles for Aβ plaques and Aβ-induced chronic neuroinflammation in the progression of AD (Choo et al., 2013; Heneka et al., 2015).

Therefore, this study focused on the multi-target effects of BIS-MEP on cognitive deficits, Aβ deposition and neuroinflammation. First, intracranial injections of the combination of Aβ and IBO were administered to establish the AD mouse model, and the Morris water maze was employed to evaluate cognitive functions. Then, immunohistochemical staining and quantitative biochemical analyses were conducted to reveal the effect of BIS-MEP on ameliorating Aβ deposition, cholinergic dysfunction and neuroinflammation (Figure 1C). The mechanism underlying its therapeutic effects will provide an important foundation for the further research and development of BIS-MEP.

## Materials and Methods

### Materials and Reagents

BIS-MEP was synthesized by the Xie lab in the Department of Chemistry, School of Pharmacy, Fudan University. IBO (I2765) and donepezil (D6821) were purchased from Sigma-Aldrich (Atlanta, GA, USA). The Aβ monoclonal antibody, 6E10, was obtained from Covance (Emeryville, CA, USA). Glial fibrillary acidic protein (GFAP) monoclonal antibody was purchased from Millipore (Temecula, CA, USA). The polyclonal antibody against ionized calcium-binding adapter molecule 1 (IBA1), a microglia-specific protein, was obtained from Arigo Biolaboratories (Taipei, Taiwan, China). Aβ_1–42_ (03112), Amplex Red Acetylcholine/AChE Assay Kit (A12217) and the Pierce BCA Protein Assay Kit (23225) were purchased from Thermo Fisher Scientific (Rockford, IL, USA). Mouse TNF-α and mouse IL-6 ELISA kits were obtained from ExCell Biology (Shanghai, China). All other reagents were purchased from commercial sources.

### Animals and Treatment

ICR mice (20–25 g, male) were supplied by the SLAC Laboratory Animal Co. Ltd. (Shanghai, China). Animals were maintained in environmentally controlled rooms (25°C, 50%–55% humidity) on a 12:12 h light-dark circle with free access to food and water. This study was carried out in accordance with the recommendations of “the Guide for the Care and Use of Laboratory Animals,” as adopted and promulgated by the United States National Institutes of Health. The protocol was approved by the the Animal Ethics Committee of Shanghai Jiao Tong University School of Medicine.

Animals were randomly assigned into five groups. (1) The hippocampus of the control group was injected with saline followed by a vehicle treatment. (2) The AβO group was administered Aβ-IBO *via* an intrahippocampal injection followed by a vehicle treatment. (3) The BIS-MEP low dose group (BIS-MEP L + AβO) was administered Aβ-IBO *via* an intrahippocampal injection followed by the BIS-MEP treatment at the dose of 0.2 μg/kg/day (s.c.). (4) The BIS-MEP high dose group (BIS-MEP H + AβO) was administered Aβ-IBO *via* an intrahippocampal injection followed by the BIS-MEP treatment at the dose of 2 μg/kg/day (s.c.). (5) The positive drug control group (donepezil + AβO) was administered Aβ-IBO *via* an intrahippocampal injection followed by the donepezil treatment at the dose of 2 mg/kg/day (i.p.; Yu et al., 2015). Drugs were administered twice a day after the intracranial injection for 21 days. The dose of BIS-MEP was adopted and modified from our previous study (Liu et al., 2013).

### Preparation of the Oligomeric Aβ_1–42_ and IBO Solution

Using a previously reported protocol (Hu et al., 2017), the lyophilized Aβ_1–42_ powder was dissolved in hexafluoro isopropanol (HFIP; H107501, Aladdin, Shanghai, China) to a concentration of 5 μg/μL at room temperature, with intermittent moderate vortexing for 1 h, and then subjected to a gentle stream of nitrogen gas to evaporate HFIP and obtain Aβ monomers. The peptide was dissolved in sterile saline at a concentration of 2 μg/μL and incubated at 4°C for 72 h to prepare Aβ oligomers. IBO was dissolved in saline at a concentration of 1 μg/μL and mixed with the Aβ oligomer solution at a ratio of 1:1 (v/v) before the intracranial injection. The final concentrations of Aβ_1–42_ and IBO were 1 μg/μL and 0.5 μg/μL, respectively.

### Intrahippocampal Injection

Aβ-IBO was injected as previously described with slight modifications (Zheng et al., 2013; Bachhuber et al., 2015). Mice were anesthetized with an intraperitoneal injection of 4% chloral hydrate (400 mg/kg) and positioned in a stereotaxic apparatus (ASI Instruments, Warren, MI, USA) to conform to the brain atlas. The Aβ-IBO solution or vehicle (sterile saline) was bilaterally injected into the hippocampus at the following coordinates: 2.3 mm posterior to the bregma, 1.8 mm lateral from the sagittal suture, and 2 mm from the surface of the skull. The injection (2 μL for each side) was performed at a rate of 1 μL/min with a microinjection syringe (Gaoge, Shanghai, China). The needle was left in place for additional 5–8 min after the injection and was then pulled back at a rate of 1 mm/min.

### Morris Water Maze

Spatial learning and memory were evaluated using the Morris water maze, as previously described (Vorhees and Williams, 2006). The experimental apparatus consisted of a circular pool (diameter: 120 cm, height: 50 cm) filled with opaque water (depth: 25 cm) at 22 ± 1°C. An escape platform (diameter: 9 cm) was placed 1 cm below the water surface. In the navigation test, mice were subjected to four trials per day for four consecutive days. The time needed to locate the hidden platform was measured as latency, and the maximum time was 60 s. The mouse that failed to locate the platform within 60 s was guided to the platform. After locating the platform, the mouse stayed on the platform for 30 s. The spatial probe test was performed on the 5th day for 60 s. The mice swam freely in the water tank without the platform and the number of times the animal crossed the platform site and distance swam in the target quadrant were recorded. All traces were analyzed using a computerized tracking system (Morris Water Maze Video Analysis System, Shanghai Yishu Software Technology Co., Shanghai, China).

### Immunohistochemistry

After the Morris water maze task, mice were anesthetized with 4% chloral hydrate (400 mg/kg, i.p.), perfused intracardially with 40 mL of ice-cold saline, and then fixed with 4% paraformaldehyde (PFA) in 0.1 M phosphate buffer. Brains were immediately removed and post-fixed with 4% PFA at 4°C in the dark. Brain tissues were successively dehydrated with 75%, 85%, 95%, and 100% alcohol solutions and embedded in paraffin wax. Ten-micrometer-thick sections were cut from paraffin blocks and then deparaffinized and microwaved in a citric acid buffer solution (10 mM, pH 6.0) for 15 min for antigen retrieval. After blocking endogenous peroxidase activity with a 3% hydrogen peroxide solution at room temperature for 15 min, sections were incubated with primary antibodies against Aβ (1:200 dilution), GFAP (1:2,000 dilution), and IBA1 (1:2,000 dilution) overnight at 4°C. Secondary antibodies conjugated with horseradish peroxidase were incubated with sections for 15 min at room temperature. Staining was detected with 3,3’-diaminobenzidine (DAB) as the chromogen and with haematoxylin prior to coverslipping. Image-Pro Plus software (Media Cybernetics, Silver Spring, MD, USA) was used to quantify areas of Aβ plaques, activated astrocytes and microglia at 40× magnification. Representative images captured at 200× magnification are shown for more detail.

### Preparation of Brain Tissue Homogenates

Mice were sacrificed by cervical dislocation followed by decapitation. The cerebral hemispheres were rapidly removed and dissected on an ice-cold plate to isolate the cortex and hippocampus. Tissues were homogenized in nine volumes of (w/v) saline. Homogenates were then centrifuged at 4,000 rpm for 10 min at 4°C. The supernatant was collected and stored at −80°C until further experimental processing. The total protein concentration in the supernatant was determined with the BCA assay according to the manufacturer’s protocol.

### Determination of Acetylcholinesterase Activity

AChE activity in the cortex and hippocampus was determined using the Amplex Red Acetylcholine/AChE Assay Kit, according to the manufacturer’s instructions (Jin et al., 2009). Briefly, cortical and hippocampal homogenates were diluted with 250 mM Tris-HCl, pH 8.0 (1:100). One-hundred microliters of sample were added to 100 μL of reaction mixture (containing 2 U/mL horseradish peroxidase, 0.2 U/mL choline oxidase, 100 μM acetylcholine and 400 μM Amplex Red reagent) and incubated for 40 min at room temperature in the dark. The formation of the fluorescent product resorufin was detected at an excitation wavelength of 571 nm and an emission wavelength of 585 nm using a Varioskan Flash multimode reader (Thermo Fisher Scientific Inc., Waltham, MA, USA).

### ELISA

The levels of proinflammatory cytokines TNF-α and IL-6 in the hippocampus and cortex were determined using standard sandwich ELISA kits, according to the manufacturer’s instructions. The absorbance was recorded at 450 nm using a microtiter plate reader, and the concentrations of TNF-α and IL-6 were calculated from standard curves.

### Statistical Analyses

All data are presented as the means ± SEM and were analyzed using SPSS software version 24 (IBM, Chicago, IL, USA). Two-way analysis of variance (ANOVA) with repeated measures was performed to analyze the Morris water maze latency and speed. Differences in Morris water maze results between groups were analyzed using one-way ANOVA followed by *post hoc* LSD multiple group comparison tests. Analyses of the results from other experiments employed Turkey’s multiple group comparison tests. *P* < 0.05 was considered a statistically significant difference.

## Results

### BIS-MEP Improves the Spatial Learning and Memory of AD Model Mice

**Figure 2:**
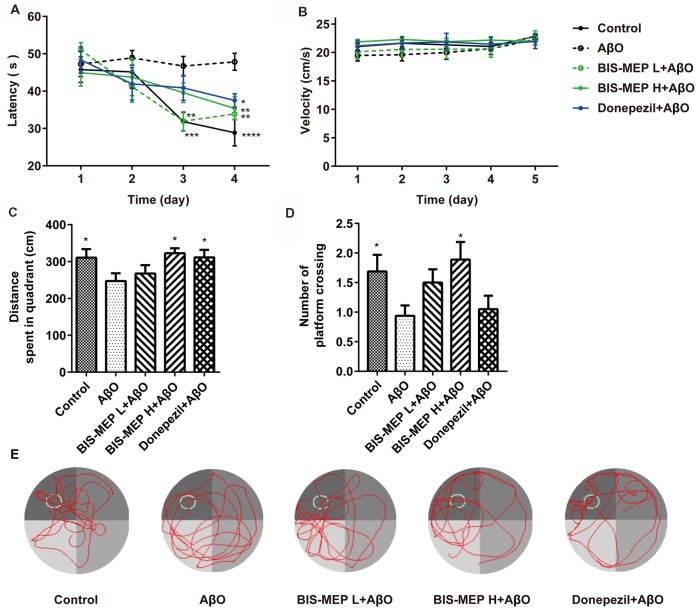
Effects of BIS-MEP on spatial reference memory in Alzheimer’s disease (AD) model mice. **(A)** The escape latency to locate the platform in the place navigation test. **(B)** The swimming velocity in the place navigation test (days 1–4) and spatial probe test (day 5). **(C)** The distance traveled in the platform quadrant in the spatial probe test. **(D)** The number of times the animals crossed the platform site in the spatial probe test. **(E)** Representative swimming paths of each group during the spatial probe test. Bars in the chart represent the means ± SEM for each group (*n* = 8–10). Significant differences between the AD model group and other groups are indicated by asterisks. *P < 0.05, **P < 0.01, ***P < 0.001, and **** *P* < 0.0001.

The Morris water maze was used to investigate the effects of BIS-MEP on spatial learning and memory impairments in mice. In the navigation test, the escape latency exhibited significant differences among groups (*F*_(4,43)_ = 6.481, *P* < 0.001) and test days (*F*_(3,129)_ = 11.909, *P* < 0.001). No significant interaction between groups and days (*F*_(12,129)_ = 1.560, *P* > 0.05) was observed. According to the results of the two-way repeated measures ANOVA, the difference among groups depended on the treatment. Vehicle-injected control mice showed significant reductions in escape latency from day to day, reflecting learning and long-term memory. Compared to the control group, mice in the AβO group displayed no changes in escape latency over the 4 training days. The difference in latencies between the two groups became significant on the 3rd (*P* < 0.01) and 4th training days (*P* < 0.0001), suggesting impaired spatial learning abilities of the AβO mice (Figure 2A). The subcutaneous injection of BIS-MEP at both the low dosage (0.2 μg/kg/day) and high dosage (2 μg/kg/day) significantly ameliorated the learning deficits induced by Aβ+IBO, as indicated by the decreased escape latency to locate the hidden platform (Figure 2A). Mice in the positive control group treated with donepezil showed a decreased escape latency as well (*P* < 0.05).

In the probe test, the one-way ANOVA analysis of distance traveled in the platform quadrant showed significant differences among groups (*F*_(4,39)_ = 2.622, *P* < 0.05). Compared to control mice, mice in the AβO group traveled a shorter distance in the target platform quadrant and crossed the platform site fewer times (*P* < 0.05). The BIS-MEP treatment (2 μg/kg/day) significantly increased the distance traveled in target quadrant (*P* < 0.05 vs. AβO) and the number of times the animal crossed the platform location (*P* < 0.05 vs. AβO). Donepezil-treated mice swam a greater distance in the target quadrant as well (*P* < 0.05; Figures 2C,D). In navigation and probe test period, no alterations in swimming speed were observed among the groups (*F*_(4,43)_ = 0.535, *P* > 0.05; Figure 2B), indicating the differences in latency and travel distance among groups were caused by Aβ oligomers injection or BIS-MEP treatment rather than mice motor deficit. According to the representative swimming paths of each group (Figure 2E), BIS-MEP-treated mice exhibited a tendency to swim around the platform site, similar to the mice in the control group, whereas AβO mice showed a characteristic circling pattern and were not prone to exploring a specific area. Based on our results, BIS-MEP effectively improved the spatial learning and memory of AβO mice.

### BIS-MEP Reverses the Cholinergic Deficits in AD Model Mice

**Figure 3:**
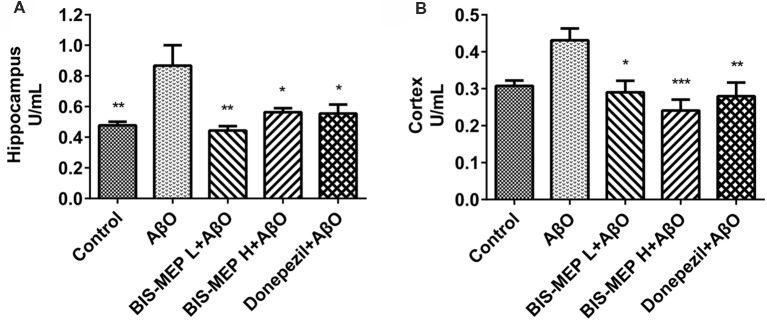
Effects of BIS-MEP on the cholinergic system in the brains of AD model mice. The acetylcholinesterase (AChE) activity in the hippocampus **(A)** and cortex **(B)** is shown in the bar graph. Bars in the chart represent the means ± SEM for each group (*n* = 5). Significant differences between the AD model group and other groups are indicated by asterisks. *P < 0.05, **P < 0.01, and ***P < 0.001.

Cholinergic deficits are highly relevant to the cognitive impairments observed in patients with AD. Therefore, AChE activity was measured in both the cerebral cortex and hippocampus to assess the effect of BIS-MEP on the function of the cholinergic system. One-way ANOVA revealed a significant effect of the treatments on enzyme activity in the hippocampus (*F*_(4,20)_ = 6.02, *P* < 0.01) and cortex (*F*_(4,20)_ = 5.763, *P* < 0.01). As shown in Figure 3, higher AChE activity was observed in the hippocampus than in the cortex. Mice in the AβO group showed a considerable increase in the AChE activity in the hippocampus (0.87 ± 0.13 U/mL, *P* < 0.01 compared with the control) and cortex (0.43 ± 0.03 U/mL, *P* < 0.05 compared with the control). The BIS-MEP treatment markedly inhibited the increase in AChE activity in both the hippocampus and cortex. The AChE activity in the high dose and low dose BIS-MEP-treated mice was decreased by 35% (*P* < 0.05 compared with the AβO group) and 49% (*P* < 0.01 compared with the AβO group) in the hippocampus and by 44% (*P* < 0.001 compared with the AβO group) and 33% (*P* < 0.05 compared with the AβO group) in the cortex. The positive drug control donepezil decreased the acetylcholinesterase activity in the hippocampus (0.55 ± 0.06 U/mL, *P* < 0.05 compared with the AβO group) and cortex (0.28 ± 0.04 U/mL, *P* < 0.01 compared with the AβO group) as well (Figures 3A,B). Thus, BIS-MEP effectively inhibited AChE activity, similar to the positive drug, consequently restoring hippocampal acetylcholine homeostasis and leading to improvements in learning and memory in AD model mice.

### BIS-MEP Reduces the Aβ Pathology in AD Model Mice

**Figure 4:**
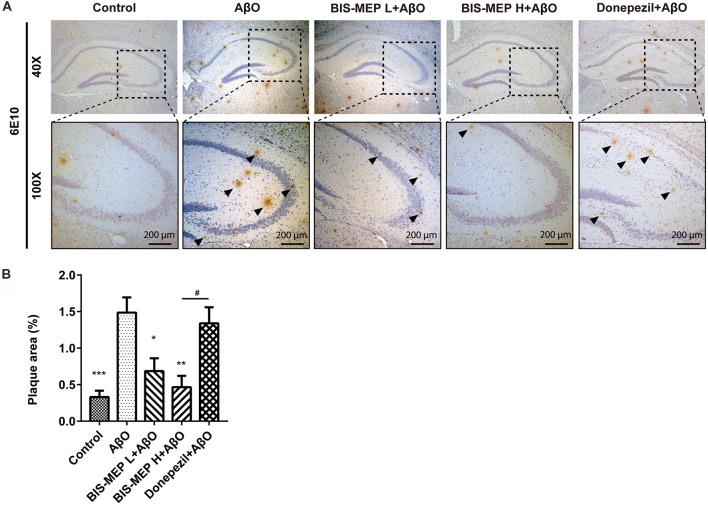
Effects of BIS-MEP on β-amyloid (Aβ) plaques in the brains of AD model mice. **(A)** Images of immunohistochemical staining for Aβ deposition in representative sections of the hippocampus from each group (40× magnification). Aβ was labeled with the 6E10 antibody. Aβ plaques are marked with black triangles. Representative images captured at 100× magnification are shown for specification. **(B)** The quantitative analysis of the percentage of Aβ plaque areas. Image-Pro Plus software was used to semi-quantitatively measure the percentage of plaque areas. Bars in the chart represent the means ± SEM for each group (*n* = 7–10). Significant differences between the AD model group and other groups are indicated by asterisks, and differences between BIS-MEP H group and Donepezil group are indicated by the pound sign. *P < 0.05, **P < 0.01, ***P < 0.001, and ^#^*P* < 0.05.

The effect of BIS-MEP on targeting both AChE activity and Aβ aggregation prompted us to examine the effects of a 3-week BIS-MEP treatment on amyloidopathy in the AD model. The Aβ plaques in the hippocampus were analyzed using immunohistochemical staining with the monoclonal antibody 6E10, which recognizes an epitope within residues 1–16 of Aβ and labels both fibrillar and non-fibrillar Aβ. Aβ plaques in hippocampus were selected (Figure 4A) and the high magnification images are shown in the lower panel of Figure 4A at 100× magnification. Treatments significantly altered the areas of Aβ plaques among different groups of mice (*F*_(4,36)_ = 9.111, *P* < 0.001). Mice in the AβO group exhibited a significant increase in the 6E10-labeled plaque burden from 0.33% ± 0.09% (control mice) to 1.49% ± 0.0.21% (percentage of the plaque area under microscope at 40× magnification; *P* < 0.001) and obvious neuronal damage in the hippocampus. In contrast, Aβ plaque areas were significantly reduced by low dose and high dose BIS-MEP treatments to 0.69% ± 0.17% (*P* < 0.05 compared with the AβO group) and 0.47% ± 0.16% (*P* < 0.01 compared with the AβO group), respectively (Figure 4B). The neuronal structure was also restored by the low and high dose BIS-MEP treatments. Donepezil slightly decreased the Aβ plaque burden and neuronal lesions compared to model group, but the difference was not significant (1.34% ± 0.22%, *P* > 0.05 compared with the AβO group). Furthermore, the effect of high dose BIS-MEP significantly exceeded donepezil (*P* < 0.05), highlighting the dual-targeting advantage of BIS-MEP. Based on these results, BIS-MEP apparently relieved the Aβ burden in the hippocampus of AβO mice.

### BIS-MEP Decreases Astrocyte and Microglia Activation

**Figure 5:**
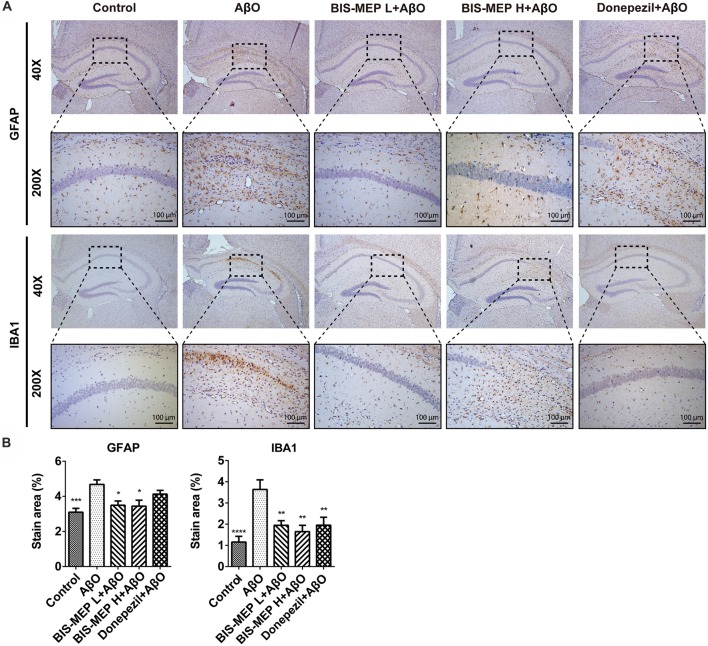
Effects of BIS-MEP on inflammatory nerve cells in the brains of AD model mice. **(A)** Images of immunohistochemical staining inflammatory biomarkers of activation in representative sections of the hippocampus from each group (40× magnification). Astrocytes and microglia were labeled with glial fibrillary acidic protein (GFAP) and ionized calcium-binding adapter molecule 1 (IBA1), respectively. Representative regions enclosed in rectangles are shown at 200× magnification for specification. **(B)** The quantitative analysis of the percentage of area occupied by activated astrocytes stained with GFAP and activated microglia stained with IBA1. Image-Pro Plus software was used to semi-quantitatively measure the percentage of activated astrocytes and microglia. Bars in the chart represent the means ± SEM for each group (*n* = 7–10). Significant differences between the AD model group and other groups are indicated by asterisks. *P < 0.05, **P < 0.01, ***P < 0.001, and ****P < 0.0001.

Astrocyte and microglia are two principle cell types involved in CNS inflammation. Misfolded and aggregated Aβ binds to the receptors on astrocytes and microglia, resulting in the release of inflammatory mediators and the induction of the innate immune response, which contribute to disease progression in subjects with AD. In the present study, immunohistochemical staining for activated astrocytes and microglia was performed using specific antibodies against GFAP and IBA1 (Figure 5A). As shown in Figure 5B, the treatments exerted significant effects on GFAP (*F*_(4,39)_ = 6.635, *P* < 0.001) and IBA1 (*F*_(4,39)_ = 7.423, *P* < 0.001) staining. The Aβ and IBO injection significantly activated astrocytes and microglia. The average GFAP-positive and IBA1-positive areas were 4.68% ± 0.26% and 3.64% ± 0.46%, respectively, and were significantly greater than those of control mice (3.10% ± 0.22%, *P* < 0.001 and 1.16% ± 0.27%, *P* < 0.001). BIS-MEP, but not donepezil, reduced the increase in GFAP staining in astrocytes to the control level. Microglia activation was significantly inhibited by the BIS-MEP treatment as the percentage of IBA1-labeled areas decreased from 3.64% ± 0.46% (AβO group) to 1.95% ± 0.22% and 1.65% ± 0.30% for the BIS-MEP L + AβO group and BIS-MEP H + AβO group, respectively (*P* < 0.01 compared with the AβO group); donepezil exerted a similar effect to BIS-MEP. Thus, BIS-MEP reduced the inflammatory reaction induced by Aβ aggregation, which may protect neurons from neurotoxic injury.

### BIS-MEP Decreases TNF-α Levels in the Brains of AD Model Mice

**Figure 6:**
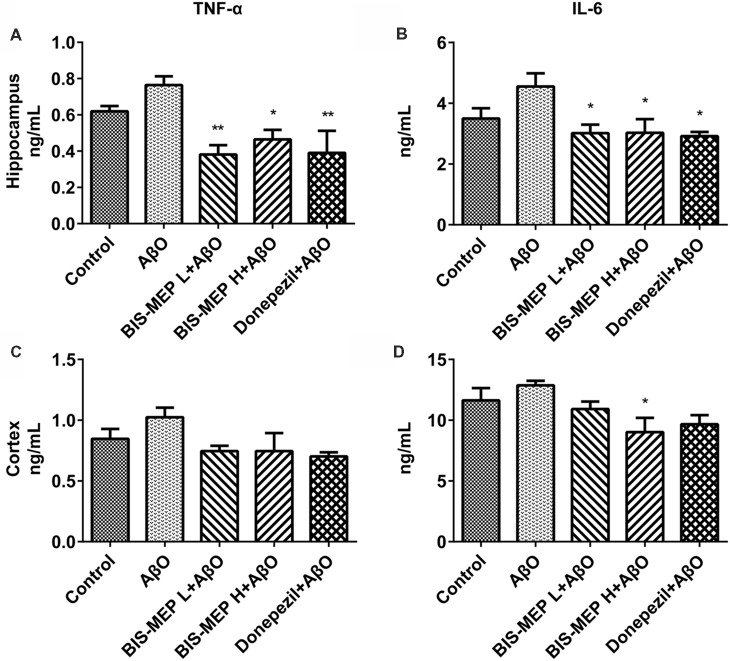
Effects of BIS-MEP on TNF-α and IL-6 levels in the brains of AD model mice. TNF-α **(A,C)** and IL-6 **(B,D)** levels in the hippocampus (upper panel) and cortex (lower panel) are shown in bar graphs. Bars in the chart represent the means ± SEM for each group (*n* = 5). Significant differences between the AD model group and other groups are indicated by asterisks. *P < 0.05 and **P < 0.01.

The levels of the pro-inflammatory cytokines TNF-α and IL-6 in the supernatants of cortical and hippocampal extracts were determined in this study to further confirm the effect of BIS-MEP on neuroinflammation. TNF-α is an important pro-inflammatory cytokine that is present at elevated levels in both the brain and plasma of patients with AD and appears to be related to disease severity. One-way ANOVA revealed significant differences in TNF-α levels in the hippocampus (*F*_(4,20)_ = 5.855, *P* < 0.01), but not in the cortex (*F*_(4,20)_ = 2.216, *P* > 0.05), between groups. Mice in the AβO group showed an increase in TNF-α levels in the hippocampal and cortical regions. After high dose and low dose BIS-MEP treatments, the levels of TNF-α were decreased by 39.2% (*P* < 0.05 compared with the AβO group) and 50.2% (*P* < 0.01 compared with the AβO group) in the hippocampus, respectively, but no significant change was observed in the cortex. The positive control drug donepezil decreased the TNF-α content from 0.77 ± 0.05 ng/mL (AβO group) to 0.39 ± 0.12 ng/mL (*P* < 0.01 compared with the AβO group) in the hippocampus and from 1.03 ± 0.08 ng/mL (AβO group) to 0.70 ± 0.03 ng/mL (*P* < 0.05 compared with the AβO group) in the cortex (Figures 6A,C).

IL-6 is another important pro-inflammatory cytokine that is present at significantly elevated levels in the brain, cerebrospinal fluid, and plasma and is particularly localized around Aβ plaques in patients with AD and animal models. Treatments exerted a significant effect on IL-6 levels in the hippocampus (*F*_(4,20)_ = 3.844, *P* < 0.05) and cortex (*F*_(4,20)_ = 3.408, *P* < 0.05). After the Aβ and IBO injection, IL-6 levels increased from 3.50 ng/mL (control group) to 4.55 ng/ml (AβO group). When treated with low dose BIS-MEP, the IL-6 level in the hippocampus was decreased to 33.7% compared with the AβO group. Furthermore, high dose BIS-MEP decreased IL-6 levels in both the hippocampus and cortex (Figures 6B,D). The donepezil treatment significantly altered IL-6 levels, consistent with a previous study (Arikawa et al., 2016). Based on these results BIS-MEP significantly decreased the production of the inflammatory cytokines TNF-α and IL-6 in the hippocampus, similar to donepezil (Hwang et al., 2010).

## Discussion

In this study, we investigated the efficacy of the multipotent AChEI BIS-MEP in classic AD model mice. Previous *in vitro* and *in vivo* studies (Xie et al., 2008; Liu et al., 2013) provided evidence that BIS-MEP inhibits AChE activity at nanomolar levels, inhibits AChE-induced Aβ aggregation and ameliorates scopolamine-induced cognitive deficits in mice. Considering the key role of Aβ in AD pathogenesis, we conducted a further study in an Aβ-induced AD mouse model. Intrahippocampal injections of Aβ oligomers combined with IBO simulated obvious amyloidopathy and cholinergic nerve injury, and subsequently caused learning and memory dysfunction (Ito et al., 2003). In a previous study, BIS-MEP (0.01, 0.1, or 1 μg/kg) was administered to mice with scopolamine-induced cognitive deficits, and BIS-MEP (0.1 μg/kg) ameliorated cognitive deficits (Liu et al., 2013). Because the half-life (T_1/2_) of subcutaneously injected BIS-MEP (0.65 mg/kg) in rats was 17.5 h (Ge et al., 2012), we administered BIS-MEP to mice twice a day to maintain the continuous effective concentration. Therefore, we injected 0.1 or 1 μg/kg BIS-MEP per treatment twice a day in the present study. After the subcutaneous injection of BIS-MEP for 21 days, the AChE activity in the hippocampus and cortex was markedly decreased in favor of the restoration of cholinergic neuron transmission. Moreover, the BIS-MEP treatment suppressed more than 50% of Aβ deposition, inhibited astrocyte and microglia activation, and decreased the release of TNF-α and IL-6 in the hippocampus, which blocked neuroinflammation and neuronal death, and more importantly, reversed the cognitive and memory deficits in the AD mouse model in the Morris water maze test.

These results revealed the multifunctional therapeutic potential of BIS-MEP to prevent or delay AD. As AD is a chronic disease with a complicated pathogenesis that is caused by multiple factors, traditional AChEIs are unable to delay disease-related deterioration. Currently, the main drug development pipelines focus on anti-amyloid, anti-tau, neuroprotective and neurotransmitter-based strategies (Cummings et al., 2017). However, anticipated drug candidates have failed in clinical trials (Cummings, 2018). The unfortunate outcomes remind us that effects on a single target are not sufficient to reverse the changes induced by the integrated and complex pathogenic network. Therefore, diverse chemical structures targeting multiple pathways involved in AD pathology have emerged (Jiao et al., 2015; Yang et al., 2017). The dual-binding AChEIs are considered valuable tools to relieve symptoms and exert disease-modifying effects (Wang et al., 2016).

As one of the standard experiments used to measure spatial learning and memory (Gandhi et al., 2000), the Morris water maze was performed in the present study. Our results confirmed that BIS-MEP significantly improved learning and memory in AD model mice. In the place navigation test, BIS-MEP significantly decreased the latency of AD model mice, which showed the potent effects of BIS-MEP on rescuing learning and memory deficits. As a potent AChEI, BIS-MEP effectively inhibited AChE activities in both the hippocampus and cortex of AD model mice. The low dose BIS-MEP treatment (0.2 μg/kg/day) exhibited a comparable effect to donepezil (2 mg/kg/day), while the dosage of donepezil was ten thousand-fold higher than that of BIS-MEP. The lower effective dose makes BIS-MEP a competitive drug candidate.

As the trigger of AD pathology, the aggregation of Aβ into senile plaques initiates a range of neurotoxic effects involved in AD etiology, such as tauopathy, oxidative stress, neuroinflammation, synaptic function abnormalities and cholinergic neuron degeneration. These factors occupy the core nodes in the AD pathogenic network (Palop and Mucke, 2010; Musiek and Holtzman, 2015; Huynh and Holtzman, 2018). In our study, immunohistochemical staining for 6E10 showed that BIS-MEP obviously alleviated Aβ deposition in the hippocampus, laying the foundation for disease intervention. According to the dual targeting property of BIS-MEP, the reduction in Aβ plaques is derived from the inhibitory effect of the BIS-MEP intervention on AChE-induced Aβ aggregation, which may account for the better performance of BIS-MEP than donepezil. Another proposed explanation is a BIS-MEP-induced decrease in Aβ production mediated by stimulating the M1 muscarinic acetylcholine receptor and subsequent regulation of the APP pathway (Fisher, 2012). These combined effects of BIS-MEP may play an important role in AD therapy, although further investigation is needed.

Moreover, BIS-MEP may inhibit the activation of microglia and astrocytes and the increase in the production of pro-inflammatory factors induced by the Aβ injection, which further relieved the inflammatory response in the nervous system. Donepezil inhibits microglial activation and thus improves learning and memory deficits in different AD mouse models (Hwang et al., 2010; Kim et al., 2014; Guo et al., 2015). Anti-inflammatory effects of donepezil on a tauopathy mouse model have also been reported (Yoshiyama et al., 2010). Likewise, BIS-MEP inhibited IBA1 expression equivalently and also decreased GFAP expression, which both contributed to the amelioration of the behavioral phenotypes. In addition to forming intercellular senile plaques, Aβ binds to microglia and astrocytes through cell-surface receptors such as CD36 and Toll-like receptors (TLR2, TLR4, and TLR6; Schwab and McGeer, 2008), activates microglia and astrocytes, stimulates the secretion of pro-inflammatory cytokines (TNF-α, IL-1β, and IL-6), mediates immune and inflammatory responses, and eventually impairs neuronal function (Sureda et al., 2006). In contrast, immune and inflammatory responses may also contribute to and driving AD pathogenesis instead of merely responding to pathophysiological changes (Heppner et al., 2015). Therefore, the anti-inflammatory effects of BIS-MEP expand its therapeutic potential in ameliorating AD pathology.

In conclusion, BIS-MEP, a multi-target inhibitor, effectively reversed the learning and memory deficits in AD model mice by inhibiting intracerebral AChE-mediated hydrolysis, reducing hippocampal AChE-chaperoned Aβ deposition, and decreasing the neuroinflammatory response. BIS-MEP represents a prospective mechanism-based AD therapeutic candidate that not only improves cognition deficits but also ameliorates the deterioration characteristic of AD pathology, which is more vital for patients with AD.

## Author Contributions

YW and HW designed the research and drafted the article. YW, JXi, MS, ZW and YZho conducted the experiments. YW, YS, JXu, LH and RZ analyzed the data and revised the article. ZQ and QX provided BIS-MEP and critically revised the article. HW, YZha and HC critically revised the article.

## Conflict of Interest Statement

The authors declare that the research was conducted in the absence of any commercial or financial relationships that could be construed as a potential conflict of interest.

## References

[B2] Alzheimer’s Association (2017). Alzheimer’s disease facts and figures. Alzheimers Dement. 13, 325–373. 10.1016/j.jalz.2017.02.001

[B1] Arikawa M. Kakinuma Y. Noguchi T. Todaka H. Sato T. (2016). Donepezil, an acetylcholinesterase inhibitor, attenuates LPS-induced inflammatory response in murine macrophage cell line RAW 264.7 through inhibition of nuclear factor kappa B translocation. Eur. J. Pharmacol. 789, 17–26. 10.1016/j.ejphar.2016.06.053 27373848

[B3] Bachhuber T. Katzmarski N. McCarter J. F. Loreth D. Tahirovic S. Kamp F.. (2015). Inhibition of amyloid-β plaque formation by α-synuclein. Nat. Med. 21, 802–807. 10.1038/nm.3885 26099047

[B4] Bond M. Rogers G. Peters J. Anderson R. Hoyle M. Miners A.. (2012). The effectiveness and cost-effectiveness of donepezil, galantamine, rivastigmine and memantine for the treatment of Alzheimer’s disease (review of Technology Appraisal No. 111): a systematic review and economic model. Health Technol. Assess. 16, 1–470. 10.3310/hta16210 22541366 PMC4780923

[B5] Choo X. Y. Alukaidey L. White A. R. Grubman A. (2013). Neuroinflammation and copper in Alzheimer’s disease. Int. J. Alzheimers Dis. 2013:145345. 10.1155/2013/145345 24369524 PMC3863554

[B6] Cummings J. (2018). Lessons learned from Alzheimer disease: clinical trials with negative outcomes. Clin. Transl. Sci. 11, 147–152. 10.1111/cts.12491 28767185 PMC5866992

[B7] Cummings J. Lee G. Mortsdorf T. Ritter A. Zhong K. (2017). Alzheimer’s disease drug development pipeline: 2017. Alzheimers Dement. 3, 367–384. 10.1016/j.trci.2017.05.002 29067343 PMC5651419

[B8] De Strooper B. Karran E. (2016). The cellular phase of Alzheimer’s disease. Cell 164, 603–615. 10.1016/j.cell.2015.12.056 26871627

[B9] Fisher A. (2012). Cholinergic modulation of amyloid precursor protein processing with emphasis on M1 muscarinic receptor: perspectives and challenges in treatment of Alzheimer’s disease. J. Neurochem. 120, 22–33. 10.1111/j.1471-4159.2011.07507.x 22122190

[B10] Galimberti D. Scarpini E. (2016). Old and new acetylcholinesterase inhibitors for Alzheimer’s disease. Expert Opin. Investig. Drugs 25, 1181–1187. 10.1080/13543784.2016.1216972 27459153

[B11] Gandhi C. C. Kelly R. M. Wiley R. G. Walsh T. J. (2000). Impaired acquisition of a Morris water maze task following selective destruction of cerebellar purkinje cells with OX7-saporin. Behav. Brain Res. 109, 37–47. 10.1016/s0166-4328(99)00160-6 10699656

[B12] Ge X. X. Wang X. L. Jiang P. Xie Y. Jiang T. Rong Z. X.. (2012). Determination of Bis(9)-(-)-Meptazinol, a bis-ligand for Alzheimer’s disease, in rat plasma by liquid chromatography-tandem mass spectrometry: application to pharmacokinetics study. J. Chromatogr. B Analyt. Technol. Biomed. Life Sci. 881–882, 126–130. 10.1016/j.jchromb.2011.12.010 22204875

[B13] Guo H. B. Cheng Y. F. Wu J. G. Wang C. M. Wang H. T. Zhang C.. (2015). Donepezil improves learning and memory deficits in APP/PS1 mice by inhibition of microglial activation. Neuroscience 290, 530–542. 10.1016/j.neuroscience.2015.01.058 25662507

[B14] Heneka M. T. Carson M. J. El Khoury J. Landreth G. E. Brosseron F. Feinstein D. L.. (2015). Neuroinflammation in Alzheimer’s disease. Lancet Neurol. 14, 388–405. 10.1016/S1474-4422(15)70016-5 25792098 PMC5909703

[B15] Heppner F. L. Ransohoff R. M. Becher B. (2015). Immune attack: the role of inflammation in Alzheimer disease. Nat. Rev. Neurosci. 16, 358–372. 10.1038/nrn3880 25991443

[B16] Hu Z. W. Ma M. R. Chen Y. X. Zhao Y. F. Qiang W. Li Y. M. (2017). Phosphorylation at Ser^8^ as an intrinsic regulatory switch to regulate the morphologies and structures of Alzheimer’s 40-residue β-amyloid (Aβ 40) fibrils. J. Biol. Chem. 292, 8846–8846. 10.1074/jbc.a116.757179 28550132 PMC5448110

[B17] Huynh T. V. Holtzman D. M. (2018). In search of an identity for amyloid plaques. Trends Neurosci. 41, 483–486. 10.1016/j.tins.2018.06.002 30053949

[B18] Hwang J. Hwang H. Lee H. W. Suk K. (2010). Microglia signaling as a target of donepezil. Neuropharmacology 58, 1122–1129. 10.1016/j.neuropharm.2010.02.003 20153342

[B19] Inestrosa N. C. Alvarez A. Pérez C. A. Moreno R. D. Vicente M. Linker C.. (1996). Acetylcholinesterase accelerates assembly of amyloid-β-peptides into Alzheimer’s fibrils: possible role of the peripheral site of the enzyme. Neuron 16, 881–891. 10.1016/s0896-6273(00)80108-7 8608006

[B20] Ito Y. Ito M. Takagi N. Saito H. Ishige K. (2003). Neurotoxicity induced by amyloid β-peptide and ibotenic acid in organotypic hippocampal cultures: protection by S-allyl-*L*-cysteine, a garlic compound. Brain Res. 985, 98–107. 10.1016/s0006-8993(03)03173-1 12957372

[B21] Jiao S. S. Yao X. Q. Liu Y. H. Wang Q. H. Zeng F. Lu J. J.. (2015). Edaravone alleviates Alzheimer’s disease-type pathologies and cognitive deficits. Proc. Natl. Acad. Sci. U S A 112, 5225–5230. 10.1073/pnas.1422998112 25847999 PMC4413288

[B22] Jin C. H. Shin E. J. Park J. B. Jang C. G. Li Z. Kim M. S.. (2009). Fustin flavonoid attenuates β-amyloid (1–42)-induced learning impairment. J. Neurosci. Res. 87, 3658–3670. 10.1002/jnr.22159 19533734

[B23] Kim H. G. Moon M. Choi J. G. Park G. Kim A. J. Hur J.. (2014). Donepezil inhibits the amyloid-β oligomer-induced microglial activation *in vitro* and *in vivo*. Neurotoxicology 40, 23–32. 10.1016/j.neuro.2013.10.004 24189446

[B24] Liu T. Xia Z. Zhang W. W. Xu J. R. Ge X. X. Li J.. (2013). Bis(9)-(-)-*nor*-meptazinol as a novel dual-binding AChEI potently ameliorates scopolamine-induced cognitive deficits in mice. Pharmacol. Biochem. Behav. 104, 138–143. 10.1016/j.pbb.2012.11.009 23262302

[B25] Musiek E. S. Holtzman D. M. (2015). Three dimensions of the amyloid hypothesis: time, space and ‘wingmen’. Nat. Neurosci. 18, 800–806. 10.1038/nn.4018 26007213 PMC4445458

[B26] Palop J. J. Mucke L. (2010). Amyloid-β-induced neuronal dysfunction in Alzheimer’s disease: from synapses toward neural networks. Nat. Neurosci. 13, 812–818. 10.1038/nn.2583 20581818 PMC3072750

[B27] Pang Y. P. Quiram P. Jelacic T. Hong F. Brimijoin S. (1996). Highly potent, selective and low cost bis-tetrahydroaminacrine inhibitors of acetylcholinesterase. Steps toward novel drugs for treating Alzheimer’s disease. J. Biol. Chem. 271, 23646–23649. 10.1074/jbc.271.39.23646 8798583

[B28] Paz A. Xie Q. Greenblatt H. M. Fu W. Tang Y. Silman I.. (2009). The crystal structure of a complex of acetylcholinesterase with a bis-(-)-*nor*-meptazinol derivative reveals disruption of the catalytic triad. J. Med. Chem. 52, 2543–2549. 10.1021/jm801657v 19326912

[B29] Schwab C. McGeer P. L. (2008). Inflammatory aspects of Alzheimer disease and other neurodegenerative disorders. J. Alzheimers Dis. 13, 359–369. 10.3233/jad-2008-13402 18487845

[B30] Sureda F. X. Gutierrez-Cuesta J. Romeu M. Mulero M. Canudas A. M. Camins A.. (2006). Changes in oxidative stress parameters and neurodegeneration markers in the brain of the senescence-accelerated mice SAMP-8. Exp. Gerontol. 41, 360–367. 10.1016/j.exger.2006.01.015 16542809

[B31] Van Dam D. De Deyn P. P. (2011). Animal models in the drug discovery pipeline for Alzheimer’s disease. Br. J. Pharmacol. 164, 1285–1300. 10.1111/j.1476-5381.2011.01299.x 21371009 PMC3229762

[B32] Vorhees C. V. Williams M. T. (2006). Morris water maze: procedures for assessing spatial and related forms of learning and memory. Nat. Protoc. 1, 848–858. 10.1038/nprot.2006.116 17406317 PMC2895266

[B33] Wang Y. Wang H. Chen H. Z. (2016). AChE inhibition-based multi-target-directed ligands, a novel pharmacological approach for the symptomatic and disease-modifying therapy of Alzheimer’s disease. Curr. Neuropharmacol. 14, 364–375. 10.2174/1570159x14666160119094820 26786145 PMC4876592

[B34] Xie Q. Wang H. Xia Z. Lu M. Zhang W. Wang X.. (2008). Bis-(-)-*nor*-meptazinols as novel nanomolar cholinesterase inhibitors with high inhibitory potency on amyloid-β aggregation. J. Med. Chem. 51, 2027–2036. 10.1021/jm070154q 18333606

[B35] Yang S.-s. Zhang R. Wang G. Zhang Y.-F. (2017). The development prospection of HDAC inhibitors as a potential therapeutic direction in Alzheimer’s disease. Transl. Neurodegener. 6:19. 10.1186/s40035-017-0089-1 28702178 PMC5504819

[B36] Yoshiyama Y. Kojima A. Ishikawa C. Arai K. (2010). Anti-inflammatory action of donepezil ameliorates tau pathology, synaptic loss and neurodegeneration in a tauopathy mouse model. J. Alzheimers Dis. 22, 295–306. 10.3233/jad-2010-100681 20847440

[B37] Yu T. S. Kim A. Kernie S. G. (2015). Donepezil rescues spatial learning and memory deficits following traumatic brain injury independent of its effects on neurogenesis. PLoS One 10:e0118793. 10.1371/journal.pone.0118793 25714524 PMC4340948

[B38] Zheng H. Zhang Y. Li W. Loh H. H. Law P. Y. (2013). NeuroD modulates opioid agonist-selective regulation of adult neurogenesis and contextual memory extinction. Neuropsychopharmacology 38, 770–777. 10.1038/npp.2012.242 23303051 PMC3671997

